# From Reconstruction to Personalization: Advances and Future Directions in Hip and Knee Arthroplasty

**DOI:** 10.3390/jcm15218732

**Published:** 2026-11-09

**Authors:** Jun-Dong Chang

**Affiliations:** Arthroplasty Center, Hallym University Dongtan Sacred Heart Hospital, Hwaseong 18450, Republic of Korea; jdchangos@gmail.com

## 1. Introduction

Total hip arthroplasty (THA) and total knee arthroplasty (TKA) are among the most successful reconstructive procedures in modern surgery, and their volume is projected to continue rising [1]. Much of this success has rested on standardized principles—conventional acetabular positioning ranges, neutral mechanical alignment in TKA, and implants designed for broad patient populations—that contributed to reproducible joint replacement. Yet a technically well-positioned implant, judged on postoperative static radiographs, does not always produce a satisfied patient [2]. Residual symptoms, instability, periprosthetic joint infection (PJI), and revision remain clinically important.

Success should be judged not only by radiographic appearance and implant survival, but also by the patient’s functional experience over time. Recognition of anatomical variation, dynamic biomechanics, and differing functional requirements has challenged the assumption that a single surgical target suits every patient.

This Special Issue, “New Advances in Hip and Knee Arthroplasty,” includes eight contributions that address this challenge from complementary perspectives. They examine spinopelvic alignment after THA, anatomical landmarks for and controllability of femoral stem anteversion, modular and anatomically adapted dual-mobility constructs, joint-line geometry in TKA, implant-retaining revision for hip infection in elderly patients, and digitally supported metabolic optimization [3,4,5,6,7,8,9,10]. Their collective value lies not in promoting a single technique, but in clarifying how anatomy, implant selection, operative strategy, and general health may be integrated into individualized care. This Editorial summarizes their principal findings, considers their implications and limitations, and outlines priorities for future research.

## 2. Dynamic Spinopelvic Function: From Static Targets to Functional Assessment

Conventional acetabular positioning relies largely on static radiographs, yet pelvic tilt alters acetabular orientation during standing, sitting, and transitional activities, and lumbar stiffness, prior spinal fusion, and aging modify these movements differently in each patient. Spinopelvic imbalance has also been identified as a cause of late dislocation after THA [11].

In a narrative review of 76 studies, Ike et al. summarized evidence that spinopelvic alignment can change after THA [3]. Early after surgery, restoration of hip motion may partially reverse hip-driven compensatory patterns, such as spinopelvic hypermobility and lumbar hyperlordosis, and several included studies reported early pelvic-tilt changes within the first three months. Over the longer term, standing pelvic tilt tends to shift posteriorly with spinal degeneration and aging; this progression has been associated with female sex, vertebral fractures, and preoperative posterior pelvic tilt.

The review also highlights the limits of prediction: it cites a model in which the prediction interval for postoperative pelvic tilt was approximately 14°, an uncertainty that may substantially narrow an individualized target zone. The functional environment of an implanted cup may therefore change over the patient’s lifetime. The authors accordingly favor risk stratification—identifying patients in whom conventional assumptions are less reliable, based on spinal alignment, pelvic mobility, contralateral hip status, and whether imbalance is hip-driven or spine-driven—over reliance on a single angular measurement. A recent systematic review likewise supported assessment of the spine–pelvis–hip complex when planning cup placement [12]. The review’s recommendation that follow-up include evaluation and management of osteoporosis is practical: vertebral fractures may influence pelvic alignment, although whether osteoporosis treatment reduces late instability after THA remains unestablished.

## 3. Femoral Stem Anteversion: Landmarks, Modifiability, and Anatomical Constraints

Femoral component version is the other component of combined anteversion, yet with cementless stems it is influenced by proximal femoral anatomy. In the combined anteversion technique, cup orientation is adjusted to the version of the stem [13]. Two studies examine how it may be estimated and to what extent it can be modified.

Yun et al. evaluated the anterior lesser trochanter line (ALTL) as a proximal landmark using computed tomography in 70 patients (140 hips) [4]. Intra- and interobserver reliability exceeded 0.81. The angle between the femoral neck axis and the ALTL correlated positively with femoral anteversion (r = 0.70) and negatively with the angle between the ALTL and the posterior condylar axis (r = −0.79). Because the posterior condylar axis is generally not directly accessible during routine THA, a reproducible proximal landmark may be useful, particularly in anterior or anterolateral approaches. However, reliable measurement on CT is not equivalent to dependable intraoperative identification; landmark visibility, soft-tissue coverage, anatomical variation, and agreement between estimated and implanted anteversion require prospective evaluation.

Yoon et al. examined rotational control in 142 hips treated with a single-wedge tapered stem and a target prosthetic anteversion of 30° [5]. In the 126 hips in which an increase was intended, mean anteversion increased from 12.72° (native) to 25.17° (prosthetic), and targeted and actual corrections correlated significantly (r = 0.633). In the 16 hips in which a decrease was intended, anteversion did not change significantly (39.13° vs. 37.84°). A lower calcar-to-canal ratio and an intended decrease were independently associated with correction failure.

These data suggest an asymmetry: with this stem, increasing anteversion was achieved more often than decreasing it, and femora with a lower calcar-to-canal ratio appeared more constrained. The findings do not justify compromising initial fixation for an angular target, and the decreased-anteversion subgroup was small. Taken together, the two studies suggest that anatomical estimation and intentional rotational adjustment are distinct but complementary considerations. Further studies are needed to determine whether integrating these approaches improves the accuracy of combined anteversion and clinical outcomes.

## 4. Dual-Mobility Constructs: Stability, Design, and Patient Fit

Dual-mobility (DM) articulation increases effective head size and jump distance, but designs differ in bearing materials, locking mechanisms, shell dimensions, and modular interfaces. A systematic review reported low dislocation rates with DM constructs in primary and revision THA [14].

Kennedy et al. compared three modular DM systems in primary THA [6]. Of 963 procedures reviewed, 297 patients with at least two years of follow-up were included in the clinical analyses (142, 110, and 45 patients), and two-year HOOS, JR scores were comparable among the implants. The reported three-year Kaplan–Meier survivorship estimates for the full cohort were 98.3%, 98.4%, and 96.9%, respectively, without a statistically significant difference among the three systems, and a newer design with an oxidized zirconium liner met the prespecified non-inferiority criterion against the two cobalt–chromium liner systems combined. These early data are reassuring, but unequal group sizes and short follow-up preclude conclusions about long-term equivalence, and metal ion levels were not assessed.

Kawate et al. evaluated a DM system designed for smaller patients, with small-diameter shells available and a 1 mm deeper center of rotation [7]. In 29 primary THAs in Japanese patients, mostly women with developmental dysplasia (Crowe I–II), cups as small as 46 mm were used, and no dislocation or intraprosthetic dislocation occurred during short-term follow-up. The mean Harris hip score improved from 39 to 89. The number of patients able to perform seiza (formal kneeling sitting) increased from 8 to 23, and those able to perform zarei (seated bowing) from 7 to 20. These culturally specific activities illustrate why outcomes should be assessed against patients’ actual daily demands. The small sample and short follow-up, however, limit conclusions about durability.

DM articulation is intended to improve resistance to dislocation, but these two studies did not establish which spinopelvic risk groups derive the greatest benefit [3,6,7]. The potential interaction between functional positioning and implant design warrants direct study, and longer-term surveillance of wear, intraprosthetic dislocation, and modular-interface complications is also needed.

## 5. Knee Arthroplasty: Reconsidering the Meaning of Alignment

In TKA, a similar tension exists between anatomical reconstruction and clinical benefit. Mechanical alignment provides a reproducible reference, whereas kinematic, restricted kinematic, and functional alignment seek to accommodate native anatomy and ligament behavior. Functional alignment, usually delivered with robotic assistance, has shown encouraging early results, although long-term studies are needed to establish its effect on implant survivorship [15]. An alignment philosophy should ultimately be judged by what patients experience, not solely by how precisely a planned angle is reproduced.

Song et al. addressed this question with a within-patient comparison [8]. Their retrospective cohort included 128 patients with bilateral posterior-stabilized TKA and a side-to-side difference of more than 3° in the change of joint-line obliquity (JLO). Mean JLO changes were 10.5° in knees with greater changes and 5.5° in knees with smaller changes. Although knees with smaller changes more often preserved their Coronal Plane Alignment of the Knee type [16] and JLO direction, preference did not differ: 40 patients expressed no preference, 44 preferred the knee with greater change, and 44 preferred the knee with smaller change. The Hospital for Special Surgery and WOMAC scores were also comparable.

The paired design partly controlled patient-level factors such as expectations and pain sensitivity, although the authors acknowledge residual confounding. They discussed variation in manual distal femoral resection as a possible contributor to differences in JLO. Their findings alone do not establish the value or cost-effectiveness of robotic or navigated TKA, should not be generalized to other designs, and do not indicate that alignment is irrelevant; technical accuracy remains important, but it should not be equated with treatment success.

## 6. Perioperative Optimization and Infection: Balancing Benefit and Physiological Burden

Outcomes also depend on the patient’s physiological condition; elevated preoperative HbA1c, for example, has been associated with an increased risk of PJI [17], making metabolic optimization an important area of investigation [9].

Ong et al. reported a retrospective pilot study of 43 patients undergoing hip or knee arthroplasty who followed a carbohydrate-restricted diet supported by meal photographs and glucose readings sent to the clinical team via WhatsApp at least four times daily [9]. Mean body weight decreased by 5.74 kg, body mass index by 2.26 kg/m^2^, and HbA1c by 0.72 percentage points. Many patients reduced or stopped glucose-lowering medication. No severe hypoglycemia or glycemia-related discharge delay occurred, and estimated glomerular filtration rate did not change significantly, although serum urea increased. In the small subgroup with lipid data, low-density lipoprotein and total cholesterol increased, which warrants further monitoring. The findings support the feasibility of the combined dietary and digital-monitoring program and document short-term metabolic changes, but they do not establish the independent contribution of digital monitoring or a reduction in postoperative complications; controlled trials with such endpoints are needed.

When PJI occurs, extraction of a well-fixed cementless femoral stem can entail long operative time, bone loss, fracture risk, and physiological stress, which are particularly relevant in elderly patients. Bahk et al. examined partial two-stage revision with retention of a well-fixed femoral stem in 28 patients aged 65 years or older (mean age, 79.5 years; mean follow-up, 46.2 months), most after hemiarthroplasty [10]. The stem was retained only when it was judged well fixed—by bone ingrowth along the stem on radiographs or by failure of gentle extraction—and when bone scintigraphy showed no uptake extending along the femoral canal and no proximal defect communicated with the distal canal. Seventeen patients completed second-stage revision, and 11 remained with a cement spacer. The authors reported infection control in all 28 patients at their respective final follow-up assessments, with initial infection control achieved in 96.4%.

These results require cautious interpretation given the retrospective design, variable follow-up, high competing mortality, and absence of a comparison group, and they do not support stem retention in all cases. Nevertheless, the study highlights the need to measure treatment burden—ambulatory status, reoperation, and mortality—alongside infection eradication. Comparative studies using standardized diagnostic criteria for PJI [18] would help clarify the indications for stem retention.

## 7. Integrating the Evidence and Future Priorities

Although the eight studies address different problems, several common principles emerge. First, anatomical measurement, surgical control, and clinical benefit are distinct. A landmark may be measured reliably without guaranteeing intraoperative accuracy, and closer reproduction of a planned alignment does not necessarily improve function or satisfaction. Second, implant performance depends on the environment in which the implant functions; hip stability reflects the combined effects of anatomy, changing spinopelvic biomechanics, component orientation, bearing design, and activity. Third, individualized treatment involves competing outcomes. Increasing femoral anteversion should not compromise initial fixation; radical component removal may facilitate eradication but impose a considerable physiological burden; and dietary intervention may improve glycemic control while altering lipid profiles. A technically achievable target is not necessarily the most appropriate clinical target. Finally, because the included studies have differing designs and limitations, findings from small or retrospective cohorts should be interpreted according to their respective populations and follow-up rather than extrapolated broadly.

Several priorities follow. First, static imaging should be complemented by dynamic and longitudinal assessment, and studies should test whether functional spinopelvic evaluation combined with navigation or robotics reduces instability. Second, intraoperative tools for femoral orientation should be validated prospectively against postoperative imaging across implants, approaches, and femoral morphologies. Third, new DM designs require long-term registry data on survivorship, intraprosthetic dislocation, wear, and metal ions, particularly for small-diameter implants and younger patients. Fourth, knee alignment research should identify which parameters influence preference, function, and implant longevity in different designs. Fifth, perioperative optimization and revision strategies for PJI should be evaluated in controlled studies with clinically meaningful endpoints, accounting for competing mortality in elderly patients. These priorities should be considered in the context of musculoskeletal aging, as osteoporosis, spinal degeneration, and declining physiological reserve may influence both primary and revision outcomes.

## 8. Conclusions

The papers in this Special Issue illustrate a shift from standardized reconstruction toward reconstruction optimized for the individual patient, spanning spinopelvic function, femoral version, implant design, knee alignment, metabolic preparation, and infection management. The challenge for future research is to determine not only what surgeons can measure or reconstruct, but which interventions provide meaningful and durable benefit for individual patients. I thank all contributing authors, the reviewers, and the editorial staff of the Journal of Clinical Medicine for their contributions to this Special Issue.
